# Early mortality on continuous renal replacement therapy (CRRT): the prairie CRRT study

**DOI:** 10.1186/s40697-016-0124-7

**Published:** 2016-07-22

**Authors:** Bhanu Prasad, Michelle Urbanski, Thomas W. Ferguson, Erwin Karreman, Nav Tangri

**Affiliations:** Section of Nephrology, Department of Medicine, Regina Qu’Appelle Health Region, 1440, 14th Avenue, Regina, S4P 0W5 Canada; College of Medicine, University of Saskatchewan, Saskatoon, S4P 0W5 Canada; Seven Oaks Hospital, 2 PD12, 2300 McPhillips Street, Winnipeg, Manitoba R2V3M3 Canada; Research and Performance Support, Regina Qu’Appelle Health Region, Regina, Saskatchewan S4P 0W5 Canada

## Abstract

**Background:**

Patients with acute kidney injury (AKI) requiring renal replacement therapy (RRT) have an increased short-term and long-term risk of mortality. In most North American intensive care units (ICUs), these patients receive continuous renal replacement therapy (CRRT).

**Objective:**

We aim to identify clinical and demographic factors associated with mortality within 24 h of initiating CRRT.

**Design:**

This paper is a prospective cohort study.

**Setting:**

The setting involves three ICUs (12-bed surgical ICU, 10-bed medical ICU, and a 7-bed combined ICU for both medical and surgical patients) of the Regina Qu’Appelle Health Region (RQHR) Saskatchewan, Canada.

**Patients:**

The patients were 106 individuals with AKI who were admitted to the ICUs and received CRRT from April 2013 to September 2014.

**Measurements:**

Date and time of admission, transfer to, and initiation of CRRT were documented. Demographic data, use of vasoactive medications, ventilator settings, pH, urine output, and chronic disease comorbidities were measured.

**Methods:**

The methods involved a stepwise multiple variable logistic regression model using death within 24 h of starting CRRT as the dependent variable, with significant variables derived from univariate analysis as covariates.

**Results:**

Of the 2634 patients admitted to the ICUs in the study period (April 2013 to September 2014), 83.6 % (2201/2634) had no AKI. Two hundred and sixty-nine or 10.2 % of the patients had stage 3 AKI. One hundred six of the 269 patients (40%) were started on CRRT. Of those on CRRT, 66/106 died in the ICU while on CRRT. Seventeen of the 66 patients (26%) died within 24 h of initiating therapy. In univariate logistic regression models, factors associated with early mortality included fraction of inspired oxygen (per 0.1 unit) (OR 1.39, 95 % CI 1.09–1.77); epinephrine dose >10 μg/min (OR 5.81, 95 % CI 1.86–18.16); vasopressin >0.02 μg/min (OR 3.99, 95 % CI 1.07–14.84); and norepinephrine dose >20 μg/min (OR 11.04, 95 % CI 2.38–51.24) which were associated with early mortality. When included in stepwise multivariate logistic regression analysis, only FiO_2_ (per 0.1 unit) and the dose of norepinephrine of >20 μg/min were independently associated with early mortality.

**Limitations:**

The small sample size was a limitation of this study.

**Conclusion:**

Patients admitted to the ICU with AKI requiring CRRT have a high risk of early mortality. In these patients, vasopressor use and hypoxia were independently associated with adverse short-term survival.

## Abrégé

**Mise en contexte:**

Les patients souffrant d’insuffisance rénale aiguë (IRA) nécessitant un traitement de suppléance rénale voient leur risque de mortalité augmenter à plus ou moins long terme. Dans la plupart des unités de soins intensifs (USI) d’Amérique du Nord, on soigne ces patients en leur prodiguant un traitement de suppléance rénale en continu (TRSC).

**Objectif de l’étude:**

L’étude visait à identifier les facteurs cliniques et démographiques associés à un décès du patient dans les 24 heures suivant l’amorce d’un TSRC.

**Cadre et type d’étude:**

Il s’agit d’une étude de cohorte rétrospective qui s’est tenue au sein de trois USI de centres hospitaliers associés au *Regina Qu’Appelle Health Region* de Regina (SK) au Canada. Les unités participantes se composaient d’une unité de soins intensifs chirurgicaux composée de 12 lits, une unité de soins intensifs médicaux comptant 10 lits et d’une USI composée de 7 lits combinant les deux types de soins.

**Patients:**

La cohorte comptait un total de 106 patients souffrant d’IRA ayant été admis à l’une ou l’autre des trois USI, et ayant reçu un TSRC entre avril 2013 et septembre 2014.

**Mesures:**

Pour chaque patient, on a consigné la date et l’heure de l’admission à l’hôpital, du transfert en USI et du moment où le TSRC a été amorcé. Les données démographiques ainsi que des renseignements cliniques tels que la prise ou non de médicaments vasoactifs, les paramètres de ventilation, la mesure du pH à l’amorce du TSRC, la diurèse et les maladies chroniques en comorbidité, lorsque présentes, ont également été enregistrées pour chaque participant à l’étude.

**Méthodologie:**

Un modèle par étapes de régression logistique à variables multiples a été utilisé pour l’analyse des données. Le décès du patient à l’intérieur des 24 heures suivant l’amorce de la dialyse en continu a servi de variable dépendante lors de l’analyse alors que les données significatives dérivant de l’analyse univariée ont été utilisées comme covariables.

**Résultats:**

Des 2634 patients admis au sein de l’une ou l’autre des USI participantes au cours de la période de l’étude, 83,6 % (2201/2634) n’étaient pas en situation d’insuffisance rénale aiguë. Un total de 269 patients (10,2 %) souffraient d’IRA de stade 3 et un TSRC a été amorcé pour 106 (40 %) d’entre eux. De ces 106 patients, 66 sont décédés à l’unité de soins intensifs, dont 17 (26 %) dans les 24 premières heures. Dans le modèle de régression logistique univariée, les facteurs associés à une mortalité précoce incluaient la fraction d’oxygène dans l’air inspiré (FiO_2_) par 0,1 unité (risque relatif [RR] : 1,39 ; intervalle de confiance [IC] à 95 % entre 1,09 et 1,77), une dose d’épinéphrine > 10 μg/minute (RR : 5,81 ; IC à 95 % entre 1,86 et 18,16), la vasopressine à > 0,02 μg/minute (RR : 3,99 ; IC à 95 % entre 1,07 et 14,84) et une dose de norépinéphrine > 20 μg/minute (RR : 11,04 ; IC à 95 % entre 2,38 et 51,24). Quant la FiO_2_ (par 0,1 unité) et la dose de norépinéphrine > 20 μg/minute, elles ont été associées de manière indépendante à de la mortalité précoce lorsqu’analysées uniquement à l’aide un modèle progressif de régression logistique multivariée,.

**Limites de l’étude:**

Le faible échantillonnage de patients limite la portée des résultats de cette étude.

**Conclusion:**

Les patients souffrant d’IRA et nécessitant un traitement de suppléance rénale qui sont admis dans les unités de soins intensifs ont un risque accru de mortalité précoce à la suite de l’amorce du traitement. On a de plus observé chez ces patients que la prise de médicaments vasopresseurs ainsi que l’hypoxie étaient deux facteurs associés de manière indépendante à des effets néfastes sur leur survie à court terme.

## What was known before

Acute kidney injury in intensive care units is associated with high short-term and long-term mortality, and severity of illness scores predict outcomes.

## What this adds

In a Canadian context, we show that mortality in the first 24 h at the start of continuous renal replacement therapy (CRRT) is 26 %. We confirm that hypoxia and vasopressor use are independent risk factors for early death.

## Background

Acute kidney injury (AKI) leading to renal replacement therapy (RRT) occurs in 5 % of intensive care unit (ICU) admissions [1–4]. In most North American ICUs, hemodynamically unstable patients with AKI requiring RRT or those with multiorgan failure are offered CRRT [5–10]. However, CRRT is resource intensive [11, 12] and may not improve outcomes for a subset of critically ill patients with AKI [13].

In particular, some patients who enter the ICU and receive CRRT and other invasive therapies such as extracorporeal membrane oxygenation may not survive more than 24 h. Patients and families often question the likelihood of survival and also the likelihood of renal survival while considering escalation of care, including CRRT [14]. Clinicians engaged in care of patients with AKI needing CRRT recognize high rates of hospital mortality and long-term mortality and low rates of complete renal recovery. This leads to challenging conversations with the families especially in the absence of definite clinical and demographic characteristics that predict early (<24 h) mortality. Lack of conclusive evidence leads to “time-limited” trials of escalation of care. Identification of these characteristics can lead to better-shared decision-making with patients and families and help clinicians frame their goals of care and prognostic discussions in the ICU [14]. Identifying predictors of early mortality with routinely measured laboratory and clinical variables would be of great assistance in appropriately identifying patients who are unlikely to benefit from initiation of CRRT.

### Objectives

In order to address this evidence gap, we performed a prospective cohort study in patients with AKI admitted to a tertiary care ICU who underwent CRRT. We were interested in both the natural history of AKI requiring CRRT and in identification of risk factors for early mortality. The primary outcome was as follows: early (24 h) mortality after CRRT initiation. Other secondary outcomes included mortality and renal outcomes post discharge for 90 days.

### Design

We conducted a prospective study in the three ICUs (12-bed surgical ICU, 10-bed medical ICU, and a 7-bed combined ICU for both medical and surgical patients) of the Regina Qu’Appelle Health Region (RQHR), Saskatchewan, Canada. The decision to initiate CRRT was made by nephrologists in consultation with intensivists, and ICU nurses implemented CRRT. The indications for starting CRRT were diuretic-resistant clinically significant edema (pulmonary edema), oliguria and/or anuria despite being adequately volume-resuscitated and resistant to diuretic use, hyperkalemia (>6.0 mmol/L), and AKI stages 2 and above on inotropic support. There are seven intensivists and six nephrologists at our site, and the decision was based on individuals rather than standardized criteria.

Patients were started on CRRT using a PRISMA dialysis machine (Hospal Gambro, St. Leonard, PQ, Canada) in continuous veno-venous hemodiafiltration (CVVHDF) mode. The initial prescription was blood flow at 150 mL/min; the dose of CRRT based on the effluent flow rates (the sum of ultra filtrate and dialysate) normalized to body weight was 35 ml/kg/h with pre-filter dilution. ST 100 and 150 filters (Hospal Gambro, St. Leonard, PQ, Canada) were based on body weights of less than or greater than 100 kg, respectively. All patients received citrate regional anticoagulation, and if there were contraindications to its use, then systemic heparin was alternatively administered.

### Patients

All patients older than 18 years of age with AKI who entered the ICUs and received CRRT were followed prospectively. Data was prospectively collected from April 2013 to September 2014. The ICU staff informed the study co-coordinator about initiation of CRRT. All patients requiring CRRT during the study period were included. Patients receiving conventional intermittent hemodialysis and patients with AKI stage 3 but not initiated on RRT were excluded. Patients with metastatic cancer and a documented evidence of dementia were not offered CRRT. There were six patients who were not on inotropes but received CRRT; they were included in the study population.

### Measurements

We created a case report form for the purpose of the study, and demographic and clinical information were obtained prospectively upon initiation of CRRT. It recorded the following: date and time of hospital admission; date and time of transfer to ICU; date and time of starting CRRT; creatinine at admission and at the start of CRRT; weight on admission to ICU and on starting CRRT; duration of oliguria and anuria prior to starting CRRT; pH values on CRRT initiation; demographics (age, sex, ethnicity); site of vascular access (right internal jugular vein, left internal jugular vein, right femoral vein left femoral vein, right subclavian vein, left subclavian vein); complications post insertion of vascular access; exposures (sepsis, critical illness, circulatory shock, burns, trauma, cardiac surgery, major non-cardiac surgery, nephrotoxic medications, and radio contrast agents); chronic disease comorbidities (congestive heart failure [CHF], hypertension, diabetes mellitus); Acute Physiology and Chronic Health Evaluation II (APACHE II) score; vasoactive support (epinephrine dose, norepinephrine dose, vasopressin dose, dopamine dose, dobutamine dose, milrinone dose); positive-end expiratory pressure (PEEP); fraction of inspired oxygen (FiO_2_); and extra corporeal membrane oxygenation (ECMO) at the initiation of CRRT. Outcomes such as ICU survival, hospital survival, and renal recovery were also recorded.

Creatinine was recorded daily for the duration of inpatient stay for all patients with AKI until discharge. For patients who had AKI, but not receiving CRRT, creatinine on admission and peak creatinine were documented. AKI was defined as per Kidney Disease Improving Global Outcomes (KDIGO) guidelines into three stages based on the increase of serum creatinine from the baseline (stage 1, 1.5 to 1.9 times the baseline; stage 2, 2.0–2.9 times the baseline; stage 3, >3 times the baseline or increase in serum creatinine to >353 μmol/L or initiation of RRT). Chronic kidney disease (CKD) was defined based on the estimated GFR recording in physician charts and pre-hospitalization creatinine values suggesting CKD as per the modified diet in renal disease (MDRD) formula. Oliguria (as defined by the criteria for AKI) was defined as urine volume of less than 0.5 mL/kg/h for 6 h or 0.3 mL/kg/h for 24 h. Renal recovery at hospital discharge was defined as follows: (1) no recovery if patients continued on RRT, (2) complete recovery if creatinine was less than 26.5 μmol/L above the baseline value, and (3) partial recovery if the creatinine level was at least 26.5 μmol/L higher than the baseline value [15]. Renal recovery, i.e., complete or partial, was assessed at time of hospital discharge. For those discharged without complete recovery of baseline renal function, renal recovery was re-evaluated at 9 months following hospital discharge.

The APACHE II scoring system, a hospital mortality prediction tool, was calculated at the point of starting CRRT, and a score of 0–100 was created for each patient. Our institutional review ethics board (REB) approved the study (REB-12-87). The data was collected confidentially and deindentified.

## Methods

Univariate analysis between groups are reported for normally or near normally distributed variables as means with standard deviations (SDs) and compared by Student’s *t* test and for non-normally distributed continuous data as medians with inter-quartile ranges (IQRs) and compared by Mann-Whitney *U* test. Categorical data are reported as proportions and compared using the chi-square test or Fisher’s exact test when appropriate.

A stepwise multiple variable logistic regression model was created, using death within 24 h as a dependent variable that considered variables which were marginally significant (*p* < 0.10) in the univariate logistic regression as covariates (APACHE scores >20, >65 years of age, sepsis, FiO_2_ per 10 % increase, epinephrine dose >10 μg/min, norepinephrine dose >20 μg/min, vasopressin dose >0.02 μg/min). Data are reported as odds ratios (ORs) with 95 % confidence intervals (CIs). Model calibration and fit were assessed by the area under the receiver operating characteristic curve (AUROC) and the Hosmer-Lemeshow goodness of fit test, respectively. A *p* value of <0.05 was considered statistically significant for all comparisons. Statistical analysis was performed using SAS Version 9.3.

## Results

### Study population

A total of 2634 patients were admitted to ICUs in the study period of April 2013 to September 2014. Of these patients, 83.6 % had no AKI, 4.6 % had stage 1 AKI, 1.7 % had stage 2 AKI, and 10.2 % had stage 3 AKI. Of the 269 patients with stage 3 AKI, only 106/269 (39 %) were started on CRRT. Of the 106 patients, 66/106 (62.5 %) died in ICU (8 died while undergoing CRRT and 58 died after CRRT was discontinued) and 2 died after leaving the ICU leading to a total in-hospital mortality of 64 % (68/106).

As shown in Table 1, the mean age ± SD of the patients is as follows: 59.15 ± 15.81 years; pH ± SD, 7.19 ± 0.15; mean norepinephrine dose ± SD, 22.5 ± 19.6; mean epinephrine dose ± SD, 11.07 ± 19.38; mean FiO_2_ ± SD was 0.64 ± 0.24; and mean APACHE II scores ± SD of 34.7 ± 8.98. Seventeen percent were post cardiac surgery, 13 % had major non-cardiac surgery, 23 % of the participants had pre-existing CKD, 49 % had hypertension, and 31 % had diabetes. The difference between clinical characteristics of the survivors and non-survivors is documented in Table 1.

**Table 1:** Overview of CRRT patients and comparison of patients who died within 24 h vs. patients who died after 24 h

Variable	Died within 24 h	Died after 24 h	All CRRT patients	*p* value
Time in hours—admission to CRRT	22.5 (10.85–101.93)	36.2 (11.62–114.92)	34.42 (11.12–114.92)	0.4925
Time in hours—ICU to CRRT	9.42 (5.18–22.5)	9.42 (3.45–28.92)	9.42 (3.6–28.92)	0.9682
Creatinine at admission (μmol/L)	156.5 (94–284)	182.5 (106.5–411.5)	180.0 (103.5–390)	0.5151
Creatinine at CRRT (μmol/L)	227 (121–370)	295 (169–413)	284.5 (165–412)	0.335
Fraction of inspired oxygen, %	0.79 (±0.21)	0.61 (±0.24)	0.64 (±0.24)	0.0075
Age	62.88 (±14.88)	58.43 (±15.96)	59.15 (±15.81)	0.2905
Duration of anuria (h)	6 (0–6)	3 (0–6)	3 (0–6)	0.7102
Duration of oliguria (h)	4 (2–8)	9 (2–12)	8 (2–12)	0.1825
Epinephrine μg/min	32.0 (±29.9)	7.07 (±13.6)	11.07 (±19.38)	<0.0001
Norepinephrine μg/min	39.4 (±23.5)	19.6 (±17.1)	22.5 (±19.6)	<0.0001
pH	7.08 (±) 0.19	7.21 (±0.14)	7.19 (±0.15)	0.0095
Vasopressin μg/min	0.03 (±0.01)	0.02 (±0.01)	0.02 (±0.01)	<0.0001
APACHE	38.5 (±9.5)	34.03 (±8.74)	34.7 (±8.98)	0.0265
Cardiac surgery	11.76 %	17.98 %	16.98 %	0.7312
Diabetes	41.18 %	29.21 %	31.13 %	0.329
Hypertension	52.94 %	48.31 %	49.06 %	0.7582
Pre-existing CKD	17.65 %	24.72 %	23.58 %	0.5291
Sepsis	52.94 %	79.71 %	60.38 %	0.4939
Sex (% male)	58.82 %	65.17 %	64.15 %	0.6172

Normally distributed variables are presented as means (±SD) and non-normally distributed variables are presented as mean (IQR). Categorical variables are presented as percentages

Seventeen out of 66 patients (26%) died within 24 h of initiating CRRT. As shown in Table 1, they had higher FiO_2_ requirements (0.79 ± 0.21 vs. 0.61 ± 0.24, *p* = 0.011), higher epinephrine doses (32.0 ± 29.9 vs. 7.07 ± 13.6, *p* = <0.0001), higher norepinephrine doses (39.4 ± 23.5 vs. 19.6 ± 14.2, *p* = <0.0001), lower arterial pH (7.08 ± 0.19 vs. 7.21 ± 0.14, *p* = 0.0091), higher vasopressin doses (0.030 ± 0.01 vs. 0.02 ± 0.01, *p* = <0. 0001), and higher APACHE scores (38.5 ± 9.5 vs. 34.03 ± 8.74, *p* = 0.0265).

### Factors associated with early mortality (<24 h) after starting CRRT

In univariate logistic regression models, factors associated with early mortality included fraction of inspired oxygen (per 0.1 unit) (OR 1.39, 95 % CI 1.09–1.77, *p* = 0.0081, area under the curve [AUC] = 0.708), epinephrine dose >10 μg/min (OR 5.81, 95 % CI 1.86–18.16, *p* = 0.0024, AUC = 0.707), vasopressin dose >0.02 μg/min (OR 3.99, 95 % CI 1.07–14.8, *p* = 0.0393, AUC = 0.642), and norepinephrine dose >20 μg/min (OR 11.04, 95 % CI 2.38–51.24, *p* = 0.0022, AUC = 0.739) were associated with early mortality (Table 2). When included in stepwise multivariate logistic regression analysis, only FiO_2_ (per 0.1 unit) and the dose of norepinephrine of >20 μg/min were independently associated with early mortality (Table 3).

**Table 2:** Univariate logistic regression predicting early mortality within 24 h

Variable	Odds ratio (95 % CI)	*p* value	C-statistic
Age over 65	1.03 (0.36–2.96)	0.9553	0.504
Pre-existing CKD (yes vs. no)	0.65 (0.17–2.48)	0.5315	0.535
APACHE score >20	1.16 (0.13–10.27)	0.8961	0.504
Epinephrine dose >10 μg/min	5.81 (1.86–18.16)	0.0024	0.707
Norepinephrine dose >20 μg/min	11.04 (2.38–51.24)	0.0022	0.739
Sepsis (yes vs. no)	0.7 (0.24–1.98)	0.4953	0.544
Vasopressin (>0.02 μg/min)	3.99 (1.07–14.84)	0.0393	0.642
High pH (<7.2)	2.35 (0.76–7.19)	0.1366	0.6
Fraction of inspired oxygen (per 0.1 unit)	1.39 (1.09–1.77)	0.0081	0.708

**Table 3:** Multivariate logistic regression model (stepwise selection)

Variable	Odds ratio (95 % CI)	*p* value
Norepinephrine dose >20 μg/min	3.37 (1.34–8.44)	0.0097
FiO_2_ per 0.1 unit	1.33 (1.1–1.62)	0.0035
Model C-statistic	0.755	

The model includes norepinephrine >20 μg/min, epinephrine >10 μg/min, FiO_2_ per 10 % increase, and vasopressin >0.02 μg/min

At 9 months follow-up, 12 out of 40 patients had died. Five out of twelve who were dialysis-dependant on discharge had died. Of the remaining 28 survivors, 17 had complete recovery of renal function to baseline and the remaining 11 had partial renal recovery.

## Discussion

In our regional cohort of critically ill patients with AKI, patients requiring CRRT had 62 % ICU mortality, with 17/66 (26 %) deaths taking place within 24 h of initiating CRRT. FiO_2_ (per 0.1 unit) and the dose of norepinephrine of >20 μg/min at the time of ICU admission were predictive of early death but with modest discrimination. Together, these findings suggest that certain high-risk features can help risk-stratify patients on CRRT, but not with sufficient accuracy to determine futility of treatment.

Our complete renal recovery rates of 60 % were similar to the Wisconsin group [16] and as reported by Schiffl et al. (56 %) [17]. Our in-hospital mortality of 64 % results were better than 75 % as reported by Stads et al. (75 %) [18], similar to other published studies [16, 17, 19, 20], but higher than 37 % by Mehta et al. [21]. The mean hospital stay for survivors in our study was less than the Wisconsin group [16] and the 72 days as published by the Taiwan group.

Given that 26 % of our patients died within 24 h, it would be useful to have better prognostic tools. Non-beneficial care can be seen as a clinical action serving no useful purpose in attaining a specified goal for a given patient. We recognize that conversations at the outset on outcomes are difficult in an intensive care environment, and we hoped to define patient characteristics that would identify a population where initiation of CRRT would have a low likelihood of benefit.

We attempted to isolate routinely collected clinical and laboratory variables that could label patients as non-beneficial prior to initiating CRRT, while trends for predictable variables were seen (higher FiO_2_ requirements while ventilated, higher norepinephrine, epinephrine and vasopressin dosage requirements, and lower arterial pH). None of the predictors identified in this study has sufficiently good predictive abilities to be used in clinical practice.

### Limitations

Our study’s strengths include its prospective design, which allowed us to carefully collect all relevant clinical data from the index hospitalization as well as pre-hospitalization records. In addition, we were able to capture all ICU admissions receiving CRRT in our region, as we are the only center to provide this service for our catchment area. However, despite this universal coverage, we were limited by our sample size of 106 patients over 14 months. Finally, we only studied patients who had AKI and received CRRT. As such, patients with AKI who may have refused ICU admission or dialysis would not be included in our cohort and could bias our findings.

## Conclusions

Patients with AKI requiring CRRT appear to be at a high risk of early mortality if they have a higher FiO_2_ and high norepinephrine >20 μg/min. However, these factors cannot reliably determine if treatment is likely to be non-beneficial, and therefore, larger multicenter studies of critically ill patients with reliable collection of clinical data are needed.
